# Novel proteomics and neuropathology of *NOTCH2NLC*-related neuronal intranuclear inclusion disease

**DOI:** 10.3389/fnagi.2026.1827292

**Published:** 2026-07-03

**Authors:** Feixia Zhan, Jiaxin Zhu, Yang Wang, Yuwen Cao, Xiya Shen, Wenlu Lv, Jiewei Wei, Xiaojun Huang, Steven X. Hou, Xinghua Luan, Li Cao

**Affiliations:** 1 Department of Neurology, Shanghai Sixth People's Hospital Affiliated to Shanghai Jiao Tong University School of Medicine, Shanghai, China; 2 Department of Genetics and Rare Diseases, Shanghai Sixth People’s Hospital Affiliated to Shanghai Jiao Tong University School of Medicine, Shanghai, China; 3 Shanghai Neurological Rare Disease Biobank and Precision Diagnostic Technical Service Platform, Shanghai, China; 4 State Key Laboratory of Genetic Engineering, Department of Cell and Developmental Biology at School of Life Sciences, Institute of Metabolism and Integrative Biology, Zhongshan Hospital, Fudan University, Shanghai, China; 5 Neurological Disorder Center, Haikou Orthopedic and Diabetes Hospital of Shanghai Sixth People's Hospital, Haikou, China

**Keywords:** neuronal intranuclear inclusion disease, neuropathology, *NOTCH2NLC* gene, protein aggregation, proteomic

## Abstract

**Background:**

Nucleotide repeat expansion disorders constitute a group of clinically and genetically heterogeneous diseases, pathologically characterized by the misfolding, aggregation, and accumulation of proteins. The expanded GGC repeats in the 5′ untranslated region (5’UTR) of the *NOTCH2NLC* gene translate into uN2CpolyG, a toxic polyglycine protein that leads to neuronal intranuclear inclusion disease (NIID). However, the precise composition of uN2CpolyG and its pathogenic mechanisms remain fully unclear.

**Objectives:**

We aimed to investigate the proteomic profile of uN2CpolyG and explore the novel neuropathology in NIID patients, which may underlie the disease pathogenesis.

**Methods:**

Mass spectrometry analysis was performed on purified intranuclear inclusions to investigate the proteomic profile. Three patients with genetically confirmed NIID were enrolled; two participants underwent skin biopsy, and one underwent brain autopsy. Skin and brain tissues derived from these patients were used to examine *NOTCH2NLC*-related pathological changes.

**Results:**

A group of enriched proteins interacting with uN2CpolyG were identified, characterized by significant intrinsically disordered regions (IDRs). Among these, we detected the co-localization of uN2CpolyG with PML and FUS in notably distinct patterns, causing significant DNA damage and impaired stress response. Furthermore, FUS- and PML-positive inclusions were confirmed in NIID patients’ tissues.

**Conclusion:**

Our findings provide novel insights into the proteomic profile and neuropathology of NIID, potentially enlightening the pathogenesis and therapeutic strategies for protein aggregation-related neurodegenerative diseases.

## 1 Introduction

Nucleotide repeat expansion disorders constitute a group of neurodegenerative diseases characterized by clinical and genetic heterogeneity, with the pathological hallmarks of protein misfolding, aggregation, and accumulation. Examples include polyglutamine (polyQ) tracts in Huntington’s disease and spinocerebellar ataxias (Lieberman et al., 2019), and dipeptide repeat proteins in familial amyotrophic lateral sclerosis and frontotemporal dementia (DeJesus-Hernandez et al., 2011). The precise mechanisms underlying the formation and toxicity of these inclusions remain incompletely understood, posing a significant challenge for clinical therapeutics. Neuronal intranuclear inclusion disease (NIID), a recently identified and rare neurodegenerative disorder, is characterized by the widespread distribution of eosinophilic, hyaline intranuclear inclusions in both the nervous system and various visceral organs (Sone et al., 2019). Clinically, the vast majority of cases are adult-onset and exhibit a highly variable phenotype, encompassing diverse combinations of cognitive impairment, cerebellar ataxia, parkinsonism, peripheral neuropathy, autonomic dysfunction, and episodic encephalopathy (Fan et al., 2022; Okubo et al., 2019; Yu et al., 2021). Following the application of skin biopsy and the identification of a GGC repeat expansion in the 5′-untranslated region (5’-UTR) of the *NOTCH2NLC* gene, the associated phenotypic spectrum has broadened to include a range of neurological disorders (Huang et al., 2022; Tai et al., 2023; Tian et al., 2022). Pathologically, the GGC repeat expansions, located within a small upstream open reading frame in the 5′ UTR (uN2C), are translated into a polyglycine-containing protein (uN2CpolyG), which serves as the primary component of the intranuclear inclusions (IIs) (Boivin et al., 2021). These IIs are not exclusive to NIID but are also present in other neurological diseases, such as fragile X-associated tremor/ataxia syndrome (Hagerman and Hagerman, 2016) and distal oculopharyngodistal myopathy (Yu et al., 2022). These disorders share considerable clinical overlap, similar pathological outcomes, and even some genetic commonalities.

To date, the detailed composition of IIs remains unclear. Electron microscopy reveals that these inclusions are round-shaped, filamentous materials lacking surrounding membranes. Located near nucleoli, they display typical p62/ubiquitin staining and do not contain other pathological proteins such as *α*-synuclein, Tau, *β*-amyloid, or TDP-43 (Mao et al., 2022; McFadden et al., 2005), suggesting that dysfunction of the protein quality control system is involved in NIID pathogenesis. In addition to p62 and ubiquitin, various non-specific proteins—such as promyelocytic leukemia (PML/TRIM19) protein (Nakano et al., 2017), SUMO molecule (Takahashi-Fujigasaki et al., 2006), and heat shock proteins (Pountney et al., 2008), ave. been found in IIs in diseases with unknown genetic etiologies. The retention of these proteins is closely related to their functions, providing potential clues to the pathogenic mechanism. To further investigate the detailed composition of uN2CpolyG, we developed constructs expressing expanded *NOTCH2NLC* with 104 GGC repeats. Mass spectrometry revealed that the proteins enriched with uN2CpolyG possess significant intrinsically disordered regions. Among these enriched proteins, FUS (Wang et al., 2024), HNRNPM (Liu et al., 2022), and PML (Takahashi et al., 2003) have been previously reported in various neurodegenerative diseases featuring cytoplasmic or nuclear inclusions. Furthermore, we detected distinct co-localization patterns of uN2CpolyG with PML and FUS, which were associated with significant DNA damage and impaired stress response. PML- and FUS-positive nuclear inclusions were subsequently confirmed in skin and brain tissues derived from NIID patients. Our findings provide a novel proteomic profile and neuropathological characteristics of NIID, shedding new light on the contribution of IIs to pathogenesis and highlighting potential avenues for therapeutic development.

## 2 Materials and methods

### 2.1 Plasmid constructs

To better recapitulate the pathological characteristics observed in NIID patients, we modified the EGFP-tagged uN2CpolyG construct (containing 104 GGC repeats) originally obtained from the Wang lab (Boivin et al., 2021). Briefly, a nuclear localization signal (SV40-NLS) was inserted at the N-terminus of the uN2C sequence, followed by fusion to EGFP, mRuby3, or an HA tag at the C-terminus. To maintain the stability of the expanded GGC repeats, all plasmids were transformed into *E. coli* (Shanghai Tsingke Biotechnology) and propagated at 37 °C. The coding sequence of FUS was amplified from a HeLa cDNA library, while the fluorescent protein sequences were PCR-amplified from pmEmerald-C1 plasmids. All final constructs were verified by Sanger sequencing.

### 2.2 Cell culture and transfection

HEK-293 cells were cultured in 6-well or 12-well plates using DMEM (high glucose) supplemented with 10% FBS, under a humidified atmosphere of 5% CO_2_ at 37 °C. For transient transfection, cells were seeded and cultured until they reached approximately 80% confluence. Plasmid DNA was then delivered into the cells using PEIpro (Polyplus) according to the manufacturer’s protocol. The culture medium was replaced 8–12 h post-transfection. Cells were harvested 36 h or 48 h after transfection for subsequent analyses.

### 2.3 Purification of intranuclear inclusions

Purification of IIs was performed as previously reported. Briefly, cells were plated in 6 cm dishes for 24 h and then transfected with SV40-NLS uN2CpolyG (GGC104)-EGFP plasmids using PEIpro. Cells were harvested 48 h post-transfection and resuspended in 10 mM Tris–HCl, pH 7.4, containing 2 mM MgCl2, 1% NP-40, 2 mM dithiothreitol (DTT), 1 mM PMSF, and complete protease inhibitor EDTA-free (MCE). Cells were ultrasonic lysed with 35% power lasting 10 s each time, with an interval of 10 s, repeated 3 times, and nuclei were collected by centrifugation at 300 g for 6 min at 4 °C, followed by resuspended in Washing buffer (10 mM Tris–HCl, pH 7.4, 5 mM MgCl2, 0.25 M sucrose, 2 mM DTT, 1 mM PMSF, and MCE) with 1% NP-40, 500 U/mL DNaseI (Beyotime), and 15 U/mL RNase A (Yeasen), mixed for 60 min at 37 °C, and centrifuged at 1500 g for 5 min. The precipitates were resuspended in Washing buffer with 2 M NaCl, mixed for 60 min at 4 °C, and centrifuged at 1,500 g for 5 min. The precipitates then were resuspended in Washing buffer with 4% sarcosyl (Sangon), mixed for 60 min at 4 °C, and centrifuged at 18,600 g for 30 min. Again, the precipitates were washed with Washing buffer with 4% sarcosyl, centrifuged at 18,600 g for 30 min, and collected as the purified inclusions component. The protein was dissolved in 1x SDS loading buffer (50 mM Tris pH 6.8, 10% glycerol, 2% SDS, 0.0012% bromophenol blue) for further mass spectrometry analysis or SDS-PAGE electrophoresis. Due to there were no intranuclear aggregates in the control group, no comparison between the uN2CpolyG and control was performed. To ensure the stability of the experiment, two independent purification-based proteomics analysis have been conducted.

### 2.4 Immunofluorescence and immunohistochemistry

For immunofluorescence of cultured cell, the post-transfected 48 h cells were fixed with 4% paraformaldehyde (PFA) for 30 min, followed by washing with 1 × PBS for 5 min, 3 times. Cells were permeabilized with 0.2% Triton X-100 in PBS for 20 min and blocked with 2% bovine serum albumin (BSA) for 1 h at room temperature. Cells then were incubated with primary antibodies diluted in 2% BSA for 1 h at room temperature, followed by PBS washing, and incubated with secondary antibodies for 1 h. After washing with PBS, cells were incubated with DAPI (2 μg/mL) for 5 min and the slides were mounted by Antifade Mounting Medium (Vector Lab).

For immunofluorescence of human skin tissues, the samples were plated on adherent slides, rewarmed to room temperature and washed with 1 × PBS three times. The tissues were fixed with 4% PFA for 30 min and permeabilized with 0.2% Triton X-100 in PBS for 20 min, then blocked with 2% BSA for 1 h at room temperature. Primary antibodies were incubated overnight at 4 °C, and the tissues were washed with PBS for 10 min, 3 times, then incubated with secondary antibodies for 1 h at room temperature, followed by PBS washing. Sections were incubated with DAPI (2 μg/mL) for 5 min and mounted with Antifade Mounting Medium. All images were taken Dragonfly microscope (Andor).

For immunohisochemistry of human skin or brain tissues, sections were fixed in 4% PFA, paraffin-embedded, cut into 8 μm thickness, and deparaffinized in xylene and rehydrated in ethanol (100–75%). The sections were performed antigen retrieval, followed by blocking with 2% BSA for 1 h at room temperature. Primary antibodies were incubated overnight at 4 °C in blocking buffer, and the sections were washed with 1 × PBS for 10 min, 3 times, incubated with secondary antibodies for 1 h at room temperature, followed by PBS washing. Then sections were incubated for 5 min in diaminobenzidine and rinsed with running water for 1 min, followed by staining cell nucleus with Hematoxylin for 3 min, and differentiating tissues with 1% hydrochloric acid alcohol solution for 10 s, and returning to blue with 1% ammonia water for 30 s. Sections were finally rehydrated in ethanol (95–100%) and permeabilized in xylene, and sealed with neutral gum after drying. Images were captured by a Zeiss AxioImager M2 microscope (Zeiss, Jena, Germany) equipped with a CCD digital camera (Vistron Systems, Tuchheim, Germany).

### 2.5 Microirradiation experiment

Twenty-four hours after transfection with Emerald-FUS with uN2cploy-mRuby3 or mRuby3, HEK-293 cells were plated onto a glass-bottom 3.5 cm dish. Then a region of interest was selected and photo-bleached with a 405 nm laser line at 100% intensity with three cycles. Adjusted the photography time and recorded all fluorescence images within 10 min after laser irradiation. The intensity of FUS at bleached sites was collected. The microarray analysis was performed on FV3000 (Olympus). Data were analyzed with Fiji/Image J.

### 2.6 Co-immunoprecipitation

Cells seeded in 6-well plates were transfected with EGFP and NLS uN2CpolyG (GGC104)-EGFP. 48 h post transfection, cells were treated with or without heat shock (HS, 43 °C) for 1 h. Then, cells were lysed in 500 μL Lysis buffer (Sangon) for 30 min followed by 3 × 5 s sonication. After centrifuge at 13,000 ×*g* for 10 min, one aliquot of the supernatants was saved for input, and the rest were incubated with 1.5 μL anti-PML stock solution (Proteintech) overnight at 4 °C. Then the cell lysates were incubated with protein A/G magnetic beads (Beyotime) at 4 °C overnight. After incubation, beads were washed 3 × 5 min with lysis buffer and suspended in 1 x SDS loading buffer (50 mM Tris pH 6.8, 10% glycerol, 2% SDS, 0.0012% bromophenol blue), boiled for 10 min at 100 °C, and analyzed by western blotting.

### 2.7 Immunoblotting

The protein samples were lysed in 1 x SDS loading buffer and boiled at 100 °C for 10 min. The samples were resolved by SDS-PAGE, and the gel were transferred to 0.45 mm PVDF membranes, followed by blocking with 5% BSA. The primary antibodies were diluted at the recommended ratio and incubated at 4 °C overnight. Blots were then incubated with HRP-conjugated secondary antibodies (1:10000, Beyotime). Bands were scanned by enhanced chemiluminescence using Western Blot Enhancer reagents (Thermo Fisher Scientific). For the immunoblot analysis of purified inclusions, the cytoplasmic protein GAPDH served as the internal loading control for the input samples. In the FUS rescue experiments, HEK293T cells were co-transfected to express uN2CpolyG-EGFP and FUS-HA at the indicated ratios. Following treatment with etoposide (ETO; 10 μM for 1 h) and a subsequent 2-h recovery period, cell lysates were analyzed by immunoblotting. The expression of phosphorylated ATM (pATM) was assessed, with the HA tag serving as a transfection control.

### 2.8 Human participants and tissues

Two patients diagnosed with NIID were recruited from the Department of Neurology at Shanghai Sixth People’s Hospital, affiliated with Shanghai Jiao Tong University School of Medicine. The diagnosis was established based on a combination of characteristic clinical manifestations and the identification of abnormal GGC repeat expansions (>60 repeats) within the *NOTCH2NLC* gene. Systematic clinical evaluations and brain magnetic resonance imaging (MRI) assessments were performed and confirmed by at least two senior neurologists to ensure diagnostic accuracy. Informed consent was obtained from all participants for the collection of skin tissue biopsies, which were harvested from the distal lateral calf (approximately 10 cm above the lateral malleolus). To serve as a control for investigating *NOTCH2NLC*-related pathological changes, a patient diagnosed with diabetic peripheral neuropathy (without GGC repeat expansions) was included. Genetic testing, including repeat-primed PCR and fluorescence amplicon length PCR, was performed to determine the GGC repeats within the *NOTCH2NLC* gene as previously described (Sone et al., 2019). The brain autopsy tissue of a patient with genetically confirmed NIID was obtained from Department of neurology, Peking University First Hospital (Wang et al., 2024). The study was approved by the ethics committee of Shanghai Sixth People’s Hospital affiliated to Shanghai Jiao Tong University School of Medicine (Approval No: 2021–219). All the participants or their guardians provided written informed consent.

### 2.9 Statistical analysis

Representative data for immunoblotting and microscopy imaging were obtained from at least two independent experiments. SPSS Statistics Version 26.0 (IBM Corp, Armonk, New York, USA) and GraphPad Prism Version 9 (GraphPad Software, San Diego, California, USA) were used to analyze the data. The difference between two independent groups were analyzed by non-parametric testing. For *p* < 0.05, differences were considered statistically significant (*p < 0.05; **p < 0.01).

## 3 Results

### 3.1 Design of constructs expressing intranuclear inclusions

**Figure 1:**
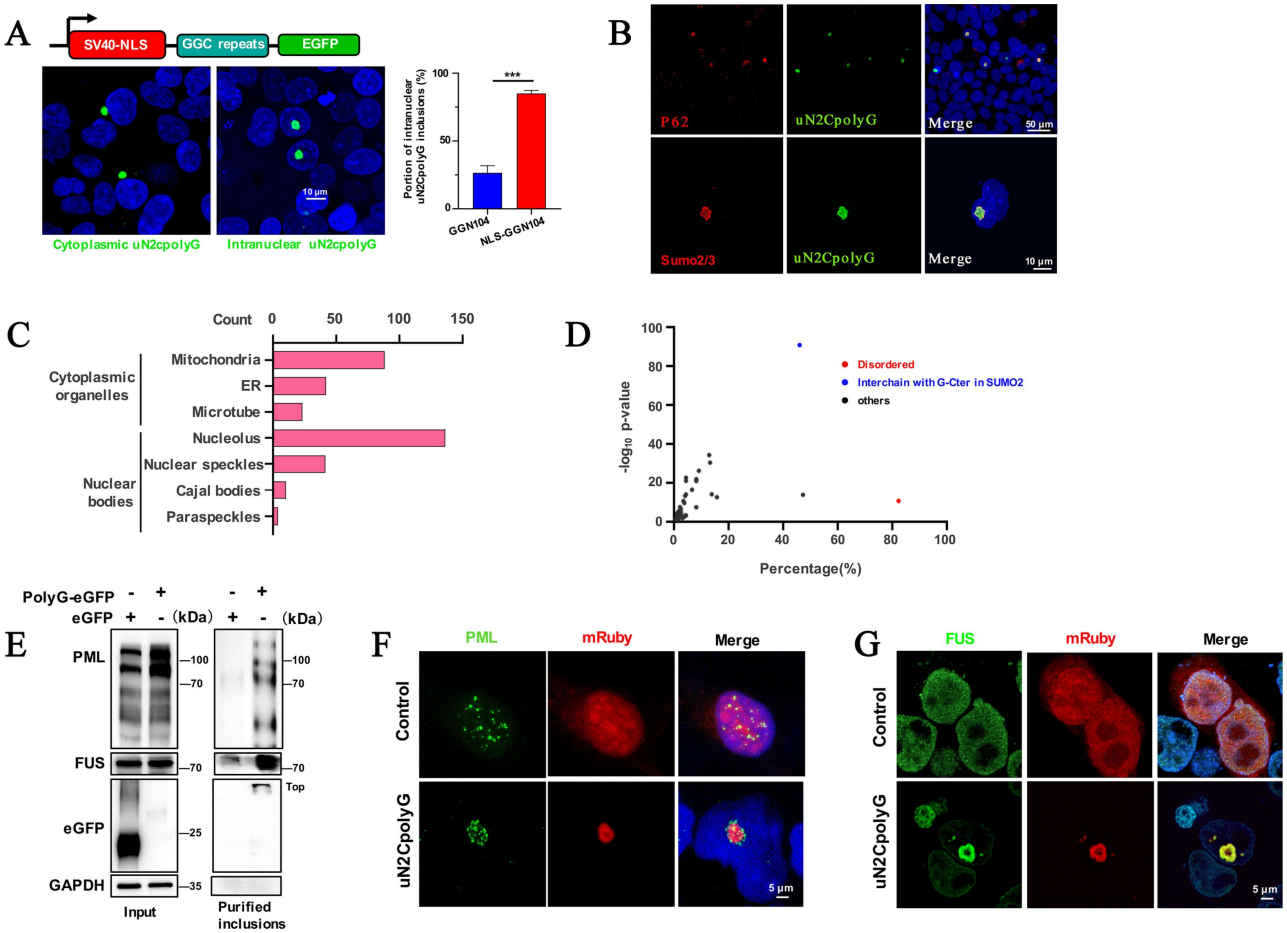
Construction of intranuclear uN2CpolyG inclusions and proteomics analysis. **(A)** Schematic representation of the plasmid constructs designed to overexpress nuclear-targeted uN2CpolyG. The SV40 nuclear localization signal (NLS) was inserted at the N-terminus of uN2C, fused with EGFP at the C-terminus. Representative fluorescence images display the formation of intranuclear inclusions, comparing constructs with and without the SV40-NLS, alongside quantification of the proportion of nuclear inclusions. Data represent analysis of n = 50 and 112 inclusions from 6 fields per group. **(B)** Representative immunofluorescence images demonstrate the co-localization of p62 and SUMO2/3 with EGFP-tagged uN2CpolyG in HEK 293 T cells. **(C)** Subcellular localization analysis indicates that the enriched proteins are closely associated with various cytoplasmic organelles and nuclear bodies. **(D)** Mass spectrometry analysis of purified uN2CpolyG aggregates reveals that the most significantly enriched proteins possess intrinsically disordered regions (IDRs), as determined by structural characteristic analysis. **(E)** Immunoblot analysis of purified inclusions confirms the direct interaction between uN2CpolyG and FUS. GAPDH, used as a cytoplasmic marker, served as the internal reference in the input control. Representative immunofluorescence images depict the distinct co-localization patterns of PML **(F)** and FUS **(G)** with mRuby3-tagged uN2CpolyG in HEK 293 T cells.

The original plasmid expressing uN2CpolyG was A gift from Wang’s lab from Department of Neurology, Peking University First Hospital. During our initial experiments involving transient transfection, we observed that the majority of aggregates formed cytoplasmic inclusions. This observation was inconsistent with the established pathology of NIID patients, particularly considering the significant differences in signal transduction and protein quality control mechanisms between the cytoplasm and nucleus. To more accurately recapitulate the pathological hallmark of NIID, specifically, the formation of intranuclear inclusions—we engineered the plasmid by inserting a nuclear localization signal (SV40-NLS) at the N-terminus of the uN2C sequence. This modification significantly increased the proportion of intranuclear inclusions (Figure 1A). Furthermore, we validated that these intranuclear uN2CpolyG aggregates exhibited strong co-localization with established landmark proteins, including P62 and SUMO2/3 (Figure 1B).

### 3.2 Proteome associated with uN2CpolyG possess intrinsically disordered regions

To further characterize the interactome of intranuclear inclusions, mass spectrometry (MS) was performed on purified uN2CpolyG aggregates. In contrast to previous studies utilizing affinity tags for immunoprecipitation, this direct analysis of purified inclusions identified over 450 unidentified proteins that were sequestered within the aggregates (Boivin et al., 2021; Liu et al., 2022). Gene Ontology biological process enrichment analysis found the enriched proteins were mainly involved in various RNA metabolism and SUMOylation modification. From the subcellular localization, these proteins were closely associated with multiple cytoplasmic organelles and nuclear bodies (Figure 1C). Due to these nuclear bodies are membraneless organelles with liquid-like properties formed by liquid–liquid phase separation, which can be driven by proteins or nucleic acids with special intrinsically disordered domains (Fonin et al., 2022; Uversky, 2017). Indeed, structural characterization analysis further confirmed that the majority of these enriched proteins contained intrinsically disordered regions (Figure 1D). Among them, we noted that FUS (97.4%), HNRNPM (61.4%), and PML (32.6%) had previously been implicated in various neurodegenerative diseases characterized by nuclear inclusions. Co-immunoprecipitation assays demonstrated a direct interaction between uN2CpolyG and FUS or PML (Figure 1E). In our cellular model, we observed distinct co-localization patterns between uN2CpolyG aggregates and PML or FUS. Specifically, PML formed a ring-like structure surrounding the uN2CpolyG aggregates (Figure 1F), whereas FUS was entirely sequestered within the inclusions (Figure 1G).

### 3.3 uN2CpolyG cause significant DNA damage and impaired stress response

**Figure 2:**
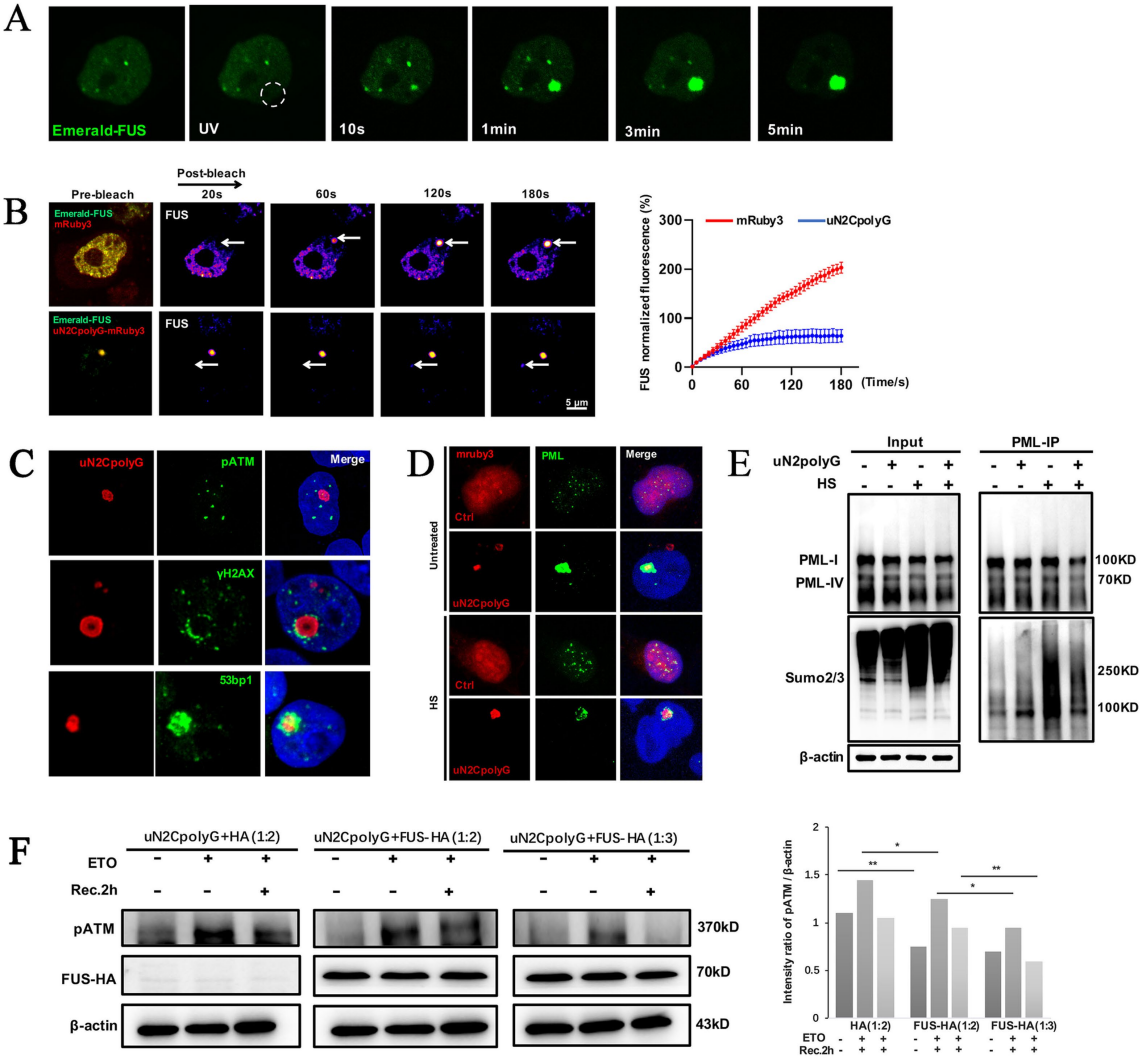
uN2CpolyG cause significant DNA damage and impaired stress response. **(A)** Time-lapse imaging shows the recruitment of Emerald-FUS to DNA damage sites in a time-dependent manner following microirradiation. **(B)** Left: Representative micrographs of cells expressing Emerald-FUS together with either mRuby3 or uN2CpolyG-mRuby3 after microirradiation. Right: Kinetic analysis of the normalized fluorescence intensity of Emerald-FUS at the irradiated site (indicated by the arrow). **(C)** Representative immunofluorescence micrographs revealing significant DNA damage in cells expressing uN2CpolyG. **(D)** Representative immunofluorescence images showing the localization of PML in HEK293 cells expressing mRuby3 or uN2CpolyG-mRuby3, treated with or without heat shock (43 °C, 1 h). **(E)** Co-immunoprecipitation analysis using anti-PML and anti-SUMO2/3 antibodies in cells expressing uN2CpolyG, with or without heat shock (43 °C, 1 h). Immunoblotting demonstrates that uN2CpolyG disrupts the PML-SUMO2/3 interaction, particularly under heat stress conditions. **(F)** Left: Immunoblot analysis of phosphorylated ATM (pATM) in HEK293T cells co-expressing uN2CpolyG-EGFP with either HA or FUS-HA at the indicated ratios, treated with etoposide (ETO, 10 μM, 1 h) followed by a 2 h recovery period. Right: Quantification of pATM levels in the lysate fractions from each group. Data are representative of two independent experiments. Statistical significance is indicated as *p.

Previous studies have shown that both PML and FUS are involved in DNA damage repair, especially the FUS dependent liquid–liquid phase separation is necessary for initiating DNA damage responses (Dellaire and Bazett-Jones, 2004; Levone et al., 2021). To investigate the cellular response to DNA damage, we utilized microirradiation to induce DNA double-strand breaks. We observed that FUS was rapidly recruited to the damage sites in a time-dependent manner (Figure 2A). However, this recruitment was significantly delayed in cells expressing uN2CpolyG compared to controls (Figure 2B). Consequently, due to this impaired recruitment, we further confirmed a significant increase in DNA damage markers—including pATM, γH2AX, and 53BP1 foci—in uN2CpolyG-expressing cells, indicating the accumulation of unresolved DNA damage (Figure 2C). Additionally, PML nuclear bodies were found to be involved in the cellular stress response under these conditions (Dellaire and Bazett-Jones, 2004). To investigate the functional impact of PML under stress conditions, we subjected cells to heat shock (HS, 43 °C, 1 h). In control cells, the number of PML nuclear bodies significantly increased following HS. However, cells expressing uN2CpolyG aggregates failed to mount this stress response, exhibiting abnormal PML distribution (Figure 2D). Furthermore, compared to controls, the interaction between PML and SUMO2/3 was dramatically disrupted in cells containing uN2CpolyG inclusions post-HS (Figure 2E), suggesting that intranuclear uN2CpolyG sequesters and incapacitates PML bodies, thereby impairing their ability to respond to cellular stress. We further found that FUS overexpression significantly reduced DNA damage (pATM foci) in a dose-dependent manner in uN2CpolyG-expressing cells (Figure 2F), indicating that the sequestration of FUS by uN2CpolyG directly contributes to its functional impairment.

### 3.4 Clinical and pathological characteristics of NIID patients

**Figure 3:**
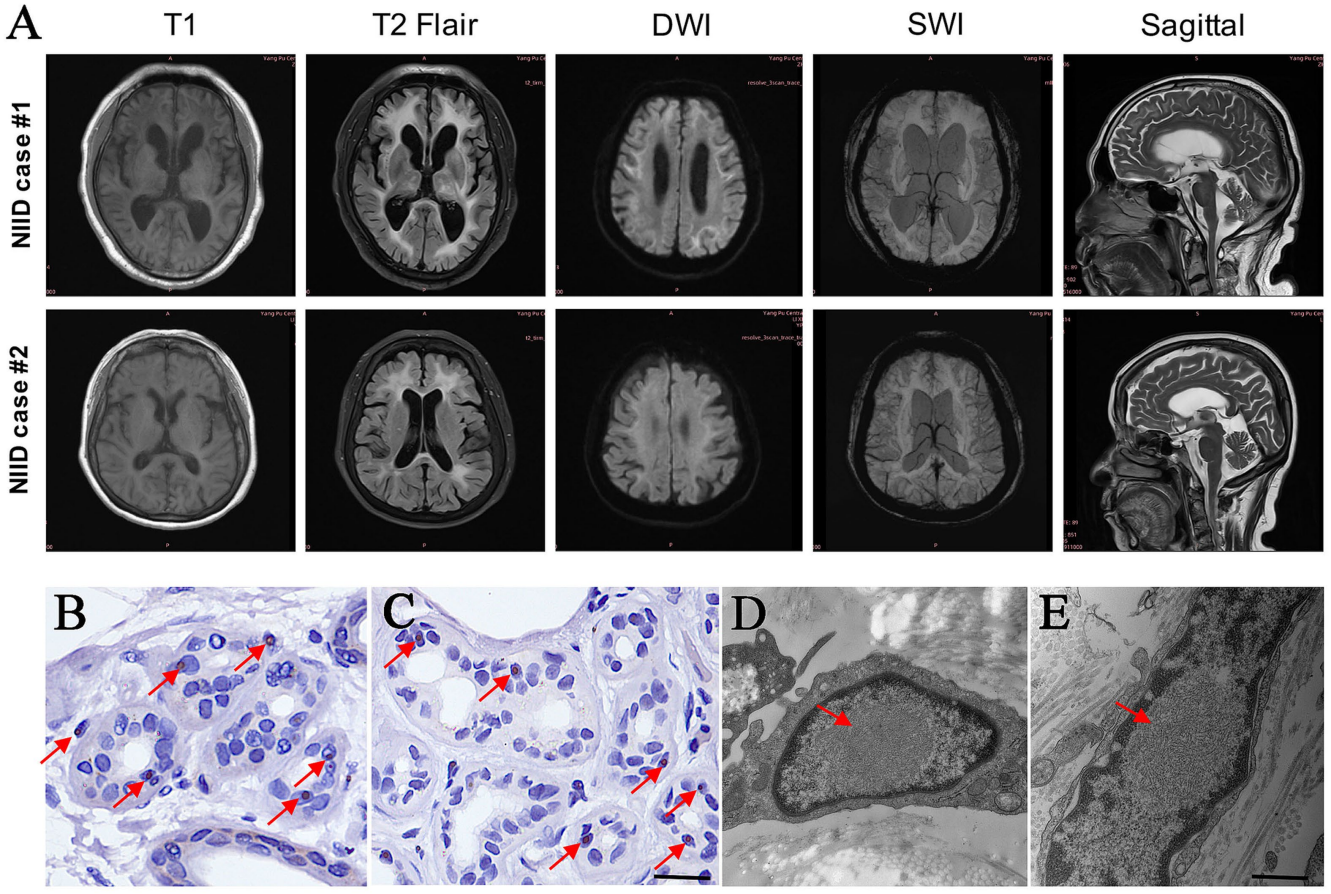
Head imaging and pathological characteristics of NIID patients. **(A)** Representative cranial imaging findings from two patients diagnosed with NIID are presented. The brain MRI abnormalities primarily manifest as hyperintense signals distributed along the corticomedullary junction on DWI sequences, accompanied by diffuse white matter lesions visible on T2WI/Flair images, as well as generalized cerebral atrophy. Notably, no microhemorrhages were detected in the SWI imaging. **(B,C)** Skin biopsies obtained from the two NIID patients revealed the presence of p62-positive intranuclear inclusions within sweat gland cells, as indicated by the arrows. **(D,E)** Electron micrographs further demonstrated the ultrastructure of these intranuclear inclusions, which are composed of disorganized filamentous arrays in the sweat gland cells (arrows). Scale bars: 20 μm (B, C); 500 nm **(D,E)**.

To further validate the interaction between uN2CpolyG and FUS or PML *in vivo*, we enrolled three patients with genetically confirmed NIID. Patient 1 (NIID case #1) was a 67-year-old male presenting with leg weakness as the initial symptom, which gradually progressed to drowsiness, memory impairment, and urinary/defecatory dysfunction. Patient 2 (NIID case #2) was a 68-year-old female with a history of recurrent stroke-like episodes accompanied by urinary difficulty and constipation. Brain MRI for both patients revealed abnormalities, primarily including high-intensity signals along the corticomedullary junction on DWI, diffuse T2WI/FLAIR white matter lesions, and cerebral atrophy (Figure 3A). Notably, no microbleeds were observed on SWI imaging. Skin pathological examinations further identified p62-positive intranuclear inclusions within sweat gland cells (Figures 3B,C). Intranuclear inclusions were captured as round-shaped filament materials without separating membranes under electron microscope (Figures 3D,E). Intranuclear inclusions were captured as round-shaped filament materials without separating membranes under electron microscope (Figures 3D,E). The third patient (NIID case#3) is a 61-year-old female with 8-year history of paroxysmal headache, and slowing progressive personality changes, abnormal behavior, cognitive decline and repeated syncope. She gradually developed to stay in bed, hypersomnia, urinary and fecal incontinence, recurrent high fever in the later stage. The patient ultimately died of respiratory failure and underwent brain autopsy. The detailed clinical, pathological, and genetic characteristics of all NIID patients are shown in Supplementary Table S1.

### 3.5 Detection of PML- and FUS-positive intranuclear inclusions in NIID tissues

**Figure 4:**
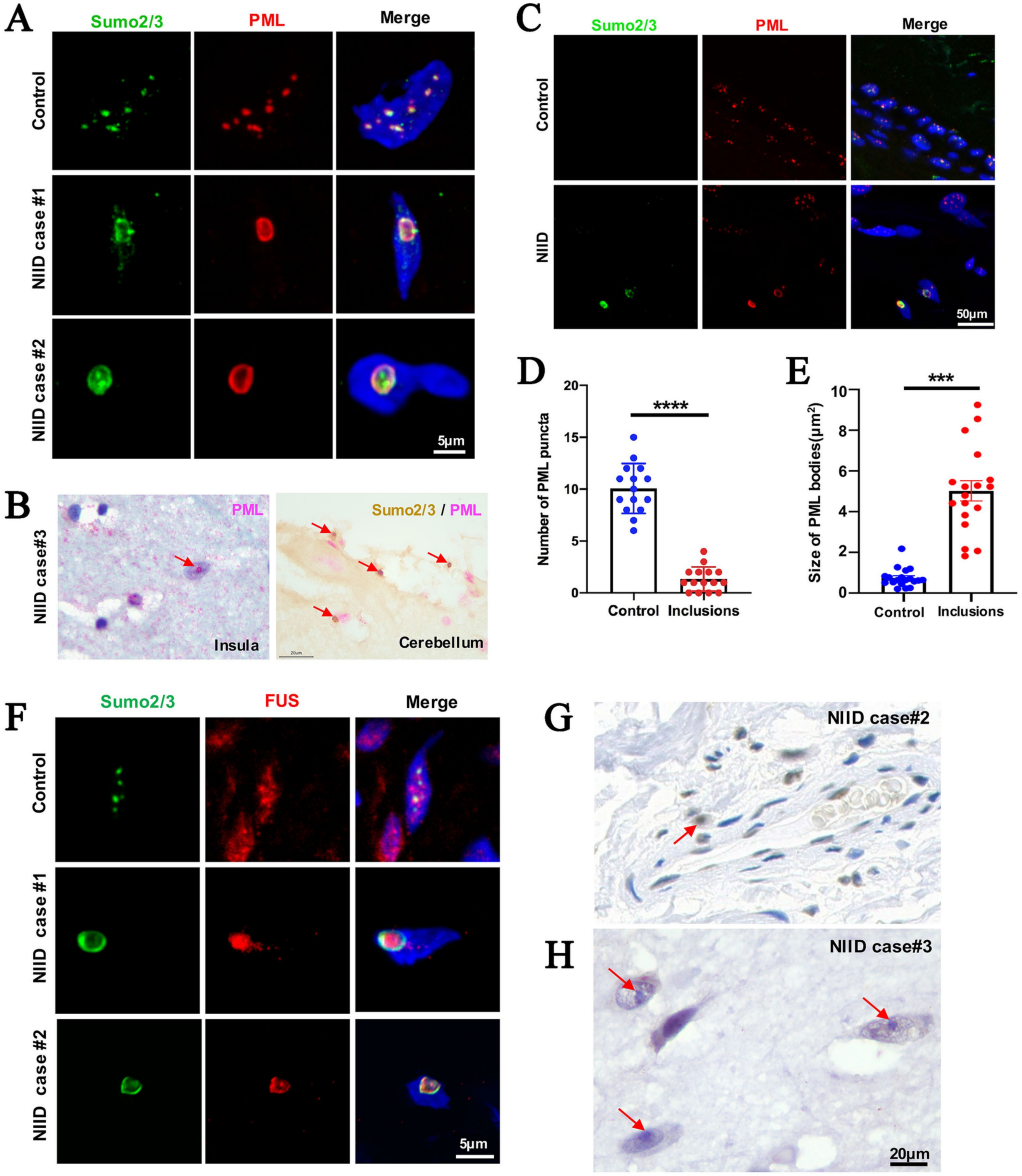
PML and FUS-positive intranuclear inclusions in NIID tissues. **(A)** Immunofluorescence analysis of SUMO2/3 and PML in skin sections from NIID patients and healthy controls. **(B)** Immunohistochemistry of PML, SUMO2/3, and PML in brain sections from NIID case #3. **(C)** Immunohistochemistry of SUMO2/3 and PML in skin sections shows a significant decrease in the number of PML nuclear bodies in cells containing intranuclear inclusions **(D)**, while their volume is significantly increased **(E)**. *n* = 13 inclusions from 7 fields were analyzed. **(F)** Immunofluorescence staining of SUMO2/3 and FUS in skin sections from NIID patients and controls. **(G)** Immunohistochemistry of FUS in skin sections from NIID case #2 and **(H)** in brain sections from NIID case #3.

We next examined the relationship between intranuclear inclusions and PML or FUS in skin and brain tissues from NIID patients. Immunofluorescence double staining for PML and SUMO2/3 revealed that PML was recruited around SUMO2/3-labeled inclusions in a ring-like structure in patient skin cells, whereas it exhibited a typical dot-like pattern in controls (Figure 4A). This specific distribution pattern was similarly confirmed in brain tissue (Figure 4B). Furthermore, compared to controls, cells containing intranuclear inclusions showed a significant decrease in the number of PML nuclear bodies alongside a marked increase in their volume, indicating that nuclear inclusions profoundly affect the morphology and properties of PML bodies (Figures 4C,E), potentially leading to functional impairment. Additionally, FUS was almost completely sequestered into intranuclear inclusions in NIID skin cells, contrasting with its dispersed nuclear distribution in the control group (Figure 4F). Immunohistochemical staining also identified several circular, FUS-immunopositive aggregates in both NIID skin (Figure 4G) and brain (Figure 4H) tissues. The presence of PML- and FUS-positive intranuclear inclusions suggests that the abnormal sequestration of these proteins plays a crucial role in the pathological mechanisms of NIID.

## 4 Discussion

NIID, a rare progressive neurodegenerative disorder, is named based on the characteristic pathology with the findings of eosinophilic nuclear inclusions in the nervous system and various visceral organs of patients (Lindenberg et al., 1968; Sung et al., 1980). Since the discovery of the GGC repeat expansion in the *NOTCH2NLC* gene, extensive efforts have been devoted to elucidating the pathogenesis of NIID; however, the underlying molecular mechanisms remain largely unknown. While intranuclear inclusions (IIs) are defined by the abnormal aggregation of diverse proteins, their specific composition is not yet fully characterized. Moreover, there is currently no definitive explanation for how uN2CpolyG proteins, which lack a canonical nuclear localization signal (NLS), enter the nucleus. The *NOTCH2NLC* gene expresses two primary transcript variants, giving rise to two distinct proteins: an early-terminated uN2CpolyG and a longer uN2CpolyG-N2C fusion protein. Whether differential expression of these isoforms in patient tissues contributes to their divergent localization patterns requires further investigation. To better model the pathological features of NIID, we modified the plasmid by inserting an SV40-NLS at the N-terminus of the uN2C sequence. MS analysis of purified intranuclear inclusions identified a specific interactome associated with uN2CpolyG. Notably, the most enriched proteins contained intrinsically disordered regions (IDRs), a key molecular feature that drives liquid–liquid phase separation (LLPS) (Mohanty et al., 2022). IDR is typically composed of low complexity domain. Proteins containing IDR are called intrinsic disordered proteins, which lack a stable three-dimensional structure, providing an structural basis for the multivalent weakly adhesive molecules interaction (Trivedi and Nagarajaram, 2022). The essence of LLPS of intracellular biomolecules is the spontaneous dynamic aggregation and separation, thereby efficiently exerting their complex and diverse biological functions (Zhang et al., 2020). Previous studies indicated that specific protein and nucleic acid components are separated by LLPS to form various membraneless organelles in the nucleus [nucleolus, Cajal bodies, nuclear plaques, perinuclear plaques, PML nuclear body (NB) and cytoplasm] (P bodies, stress granules), which participate in complex cellular biological reactions, such as RNA metabolism, transcriptional regulation, genome stability, and stress response (Fonin et al., 2022; Zhao and Zhang, 2020). Recently, studies have found that abnormal phase separation can cause protein aggregation, deposition, and denaturation, which is closely related to various neurodegenerative diseases (Ahmad et al., 2022; Patel et al., 2015; Ray et al., 2020; Solomon et al., 2021). Thus, we propose that aberrant phase transitions driven by repetitive peptides with low-complexity domains play a pivotal role in protein aggregate formation, warranting further investigation to delineate their specific contributions.

Furthermore, we observed abnormal sequestration effects mediated by uN2CpolyG. Specifically, PML encircled the uN2CpolyG aggregates in a ring-like structure, whereas FUS was completely sequestered into the inclusion core, leading to significant DNA damage and an impaired cellular stress response. PML, also known as tripartite motif-containing 19 (TRIM19), serves as a major scaffold protein for PML nuclear bodies (NBs). These membrane-less nuclear organelles play diverse roles in maintaining cellular homeostasis, including functions in apoptosis, DNA repair, gene regulation, and antiviral defense (Dellaire and Bazett-Jones, 2004). Previous study showed PML can promote the degradation of misfolded proteins through SUMO-targeted ubiquitin ligase pathway (Keiten-Schmitz et al., 2020). The loss of PML significantly exacerbate the behavior and pathological phenotype of spinocerebellar ataxia type 1 mice (Guo et al., 2014), while the aggregation of toxic polyQ proteins can be inhibited by inducing endogenous PML overexpression (Janer et al., 2006). And FUS is mainly distributed in nucleus, and can continuously shuttle between nucleus and cytoplasm (Andersson et al., 2008). As a multifunctional RNA binding protein, FUS mainly participates in the process of RNA metabolism, stress response and DNA damage repair (Gao et al., 2022; Masuda et al., 2016; Naumann et al., 2018). Mutant FUS is involved in ALS and frontotemporal lobar degeneration with the pathological findings of FUS-positive, while tau and TDP-43-negative cytoplasmic aggregates (Neumann et al., 2009). Moreover, PML or FUS-positive inclusions have also previously reported in patients with Huntington’s disease, spinocerebellar ataxia, or neuronal intranuclear hyaline inclusion disease with unknown genetic etiology (Mori et al., 2012; Nakano et al., 2017; Takahashi et al., 2003; Takahashi-Fujigasaki et al., 2006; Woulfe et al., 2010), suggesting the dysfunction of PML and FUS play a common pathogenic role in IIs-related neurodegenerative diseases. Very recently, Deng’s study showed the aberrant interaction between uN2CpolyG and FUS may lead to impaired stress granule formation and dysregulated miRNA biogenesis (Wang et al., 2024). Accumulation of DNA damage and abnormal stress response are believed to induce cell death and/or protein dysfunction, leading to chronic inflammation, which is an important risk factor for promoting disease or aging (Wang et al., 2021). Therefore, our identification of PML- and FUS-positive inclusions in NIID patient tissues further corroborates their critical pathogenic role. Further investigation is required to elucidate the common molecular mechanisms underlying the dysfunction of proteins sequestered by IIs, paving the way for potential therapeutic strategies for these disorders.

In summary, our study demonstrates that proteins enriched in uN2CpolyG inclusions contain prominent IDRs, and that abnormal uN2CpolyG sequestration induces significant DNA damage and impairs the stress response. Furthermore, we provide novel neuropathological insights into NIID. These findings may illuminate new avenues for understanding the pathogenesis and developing potential therapies for protein aggregation-related neurodegenerative diseases.

## Data Availability

The original contributions presented in the study are included in the article/Supplementary material, further inquiries can be directed to the corresponding authors.
