# Supplementary material for: Novel proteomics and neuropathology of NOTCH2NLC-related neuronal intranuclear inclusion disease
Source: Front Aging Neurosci. 2026 Jul 3;18:1827292. doi: 10.3389/fnagi.2026.1827292 (PMC13375458; doi:10.3389/fnagi.2026.1827292)
Supplement: Supplementary file 1 [file Table_1.DOCX]

**Supplementary Table S1: Clinical, pathological, and genetic characteristics of NIID patients**

| **Patient ID** | **Sex/Age(y)** | **AAO(y)** | **Family history** | **Initial symptoms** | **Other manifestations** | **Brain MRI** | **Skin biopsy** | **GGC repeats** |
| --- | --- | --- | --- | --- | --- | --- | --- | --- |
| NIID case#1 | M/68 | 67 | Negative | Weakness in legs | Drowsiness, memory impairment，urinary and fecal problems | + | p62+ | 108 |
| NIID case#2 | F/69 | 68 | Negative | Stroke-like episodes | Limb weakness, dizziness, difficulty urinating, constipation | + | p62+ | 113 |
| NIID case#3 | F/69 | 61 | Positive | Paroxysmal headache and abnormal behavior | Personality changes，cognitive decline，repeated syncope，difficulty urinating，fecal incontinence, ultimately died of respiratory failure | + | p62+ | 132 |

**Abbreviations:** NIID: neuronal intranuclear inclusion disease; y: year; AAO: age at onset; MRI: magnetic resonance imaging; M: male; F: female.

**Note:** The abnormalities in brain MRI mainly include high-intensity signals along the corticomedullary junction on DWI and diffuse T2WI/Flair white matter lesions.
